# Interplay between pathogen-mediated immune evasion and innate immune negative regulation in gastrointestinal tumors

**DOI:** 10.3389/fimmu.2026.1951036

**Published:** 2026-09-08

**Authors:** Mengxi Shi, Yihua Zeng, Chao Chen, Xiaofei Zuo

**Affiliations:** 1 Department of Anesthesiology, Sichuan Provincial People’s Hospital, University of Electronic Science and Technology of China, Chengdu, China; 2 Department of Respiratory and Critical Care Medicine, West China Hospital, Sichuan University/West China School of Nursing, Sichuan University, Chengdu, Sichuan, China; 3 Department of Thyroid Surgery, Sichuan Provincial People’s Hospital, University of Electronic Science and Technology of China, Chengdu, China; 4 Department of Gastrointestinal Surgery, Sichuan Provincial People’s Hospital, University of Electronic Science and Technology of China, Chengdu, China

**Keywords:** chronic inflammation, gastrointestinal tumors, innate immune reprogramming, negative immune regulation, pathogens, innate immunity

## Abstract

Persistent colonization by gastrointestinal pathogens is an important environmental factor contributing to the development and progression of gastric and colorectal cancer. *Helicobacter pylori* (*H. pylori*), Epstein-Barr virus (EBV), *Fusobacterium nucleatum* (*F. nucleatum*), enterotoxigenic *Bacteroides fragilis* (ETBF), and pks-positive *Escherichia coli* (*E. coli*) can induce chronic inflammation and reshape the tumor microenvironment via virulence factors, epithelial barrier disruption, DNA damage, and immune dysregulation. Conventional views have largely focused on the tumor-promoting effects of chronic inflammation; however, this view does not fully explain the coexsistence of persistent inflammation and immune evasion during chronic infection. In this review, we proposed that the key feature of pathogen-associated inflammation is not simply its intensity but rather the imbalance between sustained inflammatory stimulation and insufficient immune elimination. Rather than treating pathogen-mediated immune evasion, host-derived innate immune negative regulation, and adaptive immune checkpoint suppression as parallel mechanisms, this review examined how these functionally distinct layers converge in gastrointestinal tumors. Pathogen immune evasion allows persistent microbial stimulation, host negative-feedback pathways dampen PRR- and interferon (IFN)-associated signaling, and checkpoint-mediated suppression further impairs cytotoxic clearance. Together, these processes create a state in which sustained inflammation coexists with insufficient immune elimination. This state can impair antigen presentation, cytotoxic immune responses, and pathogen clearance, thereby promoting immune tolerance and tumor progression. Understanding this mechanism may offer a conceptual basis for pathogen control, innate immune reprogramming, and precision therapeutic intervention in gastrointestinal tumors.

## 1 Introduction

Gastric cancer and colorectal cancer are major gastrointestinal malignancies, representing a substantial global disease burden. Several decades ago, gastric cancer was among the most frequently diagnosed cancers globally. In recent years, both its incidence and mortality have declined in some regions, largely due to improvements in lifestyle, food preservation, *Helicobacter pylori* (*H. pylori*) control, and screening efforts (1, 2). Nevertheless, according to GLOBOCAN 2022, gastric cancer still ranks as the fifth most commonly diagnosed cancer and the fifth leading cause of cancer-related death globally (3, 4). Colorectal cancer is also highly prevalent globally, with its development closely linked to age, dietary patterns, obesity, metabolic disorders, genetic susceptibility, gut microbiota dysbiosis, and chronic inflammation (3, 5, 6). Despite advances in screening, early diagnosis, and multimodal therapy, the mechanisms underlying gastric and colorectal cancer remain complex and poorly understood (7, 8). Among these mechanisms, the interplay among pathogens, chronic inflammation, and the tumor immune microenvironment is increasingly recognized as a key driver of gastrointestinal tumorigenesis and progression. In this review, we focused on gastric and colorectal cancers as representative models in which pathogen-associated carcinogenesis and mucosal immune remodeling have been extensively characterized.

The gastrointestinal mucosa is continuously exposed to commensal microbiota and exogenous pathogens, making it a key site for infection-associated tumorigenesis (9). *H. pylori* is the most representative pathogen in this context, accounting for approximately 89% of non-cardia gastric cancers through chronic infection, which underscores its central role in gastric carcinogenesis (10, 11). Epstein-Barr virus (EBV)-associated gastric cancer accounts for approximately 10% of all gastric cancers (12) and is frequently characterized by immune cell infiltration and PD-L1 upregulation. In colorectal cancer, *Fusobacterium nucleatum* (*F. nucleatum*), enterotoxigenic *Bacteroides fragilis (ETBF)*, and pks-positive *Escherichia coli* (*E. coli*) do not act as single dominant etiological agents, yet they are repeatedly detected in tumor tissues and each is associated with distinct effects—immune suppression, barrier disruption, inflammation induction, and DNA damage, respectively (13–15). These pathogens are therefore not mere bystanders within the tumor ecosystem; instead, they may contribute to gastrointestinal tumor initiation and progression via persistent colonization, release of virulence factors, and remodeling of the local immune ecology.

Persistent pathogen colonization directly drives sustained mucosal inflammation at the tissue level. Pathogen-associated molecular patterns (PAMPs) can be recognized by pattern-recognition receptors (PRRs), including Toll-like receptors (TLRs), NOD-like receptors (NLRs), RIG-I-like receptors (RLRs), and the cGAS-STING pathway (16–20). These signals trigger downstream NF-κB, MAPK, IFN regulatory factor (IRF), and JAK-STAT pathways, which drive the production of inflammatory mediators such as IL-1β, IL-6, TNF, chemokines, and type I interferons (IFNs) (21–24). During acute infection, these responses help clear pathogens and promote tissue repair (25). However, with persistent infection, protective antimicrobial inflammation may evolve into chronic non-resolving inflammation (26–28). Accumulating evidence indicates that chronic inflammation is a major risk factor for tumorigenesis, as it promotes aberrant epithelial proliferation, oxidative stress, DNA damage, angiogenesis, stromal remodeling, and immune suppression (24, 29, 30). Thus, in gastrointestinal tumors, pathogens are not merely exogenous inflammatory stimuli but rather active drivers of chronic inflammation and tumor microenvironment remodeling (31, 32).

However, the concept that “chronic inflammation promotes cancer” alone is insufficient to explain pathogen-associated gastrointestinal tumorigenesis. A central unresolved question is why persistently colonizing pathogens can continue to induce inflammation yet evade immune clearance, and why sustained inflammation fails to effectively translate into antigen presentation, cytotoxic killing, and antitumor immunity. These observations suggest that the defining feature of pathogen-associated inflammation is not simply an increase in inflammatory intensity, but rather an imbalance between persistent inflammatory stimulation and inadequate immune clearance (33). This imbalance sustains a prolonged state in the gastrointestinal mucosa, driven by pathogen-derived stimulation, chronic inflammation, aberrant repair, and immune tolerance, which ultimately promote tumor initiation and progression (34). In this framework, pathogen-mediated immune evasion serves as an upstream driver of persistent stimulation, host-derived negative regulation provides inhibitory feedback on innate immune signaling, and checkpoint-mediated suppression acts as a downstream effector mechanism that further limits immune clearance.

## 2 Persistent pathogen colonization as a trigger for gastrointestinal tumor-associated inflammation

The gastrointestinal tract is one of the most extensively exposed mucosal interfaces between the human body and the external microbial environment. Persistent pathogen colonization is therefore regarded as an important environmental driver in gastrointestinal tumorigenesis. Epidemiological evidence, animal models, and multi-omics studies have shown that certain pathogens not only induce chronic inflammation but also promote the development of gastrointestinal tumors by disrupting host immune homeostasis, compromising mucosal barrier integrity, and reshaping the local tissue microenvironment.

### 2.1 Gastrointestinal pathogen colonization and the establishment of a persistent inflammatory microenvironment

In gastric cancer, *H. pylori* is currently the most well-established infectious risk factor. Its virulence factors [e.g., CagA, VacA, and low-immunogenic lipopolysaccharide (LPS)] can chronically stimulate the gastric mucosal epithelium and induce persistent inflammatory responses. As a result, the gastric mucosa is kept in a state of repeated injury and aberrant repair, progressing through the classical pathological sequence known as the Correa cascade: chronic active gastritis, atrophic gastritis, intestinal metaplasia, dysplasia or intraepithelial neoplasia, and ultimately gastric cancer (35–37). During this process, the sustained inflammatory microenvironment is characterized by enhanced oxidative stress, DNA damage accumulation, epigenetic remodeling, and immune escape, all of which collectively promote the malignant transformation of gastric epithelial cells (38).

By contrast, colorectal cancer is characterized by more prominent gut microbiota dysbiosis. Recent studies have identified *F. nucleatum*, ETBF, and pks-positive *E. coli* as key pathogenic bacteria closely linked to colorectal cancer development and progression. *F. nucleatum* promotes tumor cell adhesion, activates oncogenic signaling, and suppresses antitumor immune responses via the adhesin FadA and the immunomodulatory protein Fap2 (39). Clinically, increased *F. nucleatum* abundance in colorectal tumor or stool samples has been investigated for CRC detection and prognostic assessment, while mechanistic studies have associated it with chemotherapy resistance and context-dependent responses to PD-1 blockade. Experimental depletion of tumor-associated *Fusobacterium* can has been shown to reduce tumor growth, further supporting its potential as a therapeutic target (40–45). ETBF secretes *Bacteroides fragilis* toxin (BFT), which disrupts intestinal epithelial barrier integrity and drives persistent inflammatory responses (46). pks-positive *E. coli* produces colibactin, a genotoxin that induces DNA double-strand breaks, genomic instability, and tumorigenic mutations (47–49).

In addition to bacteria, viral infection also drives the development and progression of gastrointestinal tumors. EBV-associated gastric cancer accounts for approximately 10% of all gastric cancers and is typically marked by abundant immune cell infiltration, high PD-L1 expression, and a distinct inflammatory tumor microenvironment (50–52). Persistent EBV latency not only sustains inflammatory stimulation but also facilitates tumor immune escape by modulating host antiviral immunity, antigen presentation, and immune checkpoint molecule expression. Increasing evidence thus suggests that pathogens are not passive bystanders within tumor tissues but active drivers of chronic gastrointestinal inflammation, microenvironment remodeling, and tumor progression.

The contribution of these pathogens to gastrointestinal tumorigenesis differs markedly, depending on their biological characteristics and carcinogenic mechanisms. *H. pylori* is closely associated with long-term gastric colonization, virulence-factor-mediated epithelial injury, chronic inflammation, and progression along the Correa cascade (10, 35–38). EBV-associated gastric cancer, by contrast, features viral latency, non-coding RNA dysregulation, epigenetic alterations, IFN-related immune modulation, and immune checkpoint activation (8, 53). In colorectal cancer, *F. nucleatum*, ETBF, and pks-positive *E. coli* are more closely linked to dysbiosis and tumor-associated microbial enrichment, each contributing through distinct mechanisms—immune suppression, epithelial barrier disruption, inflammatory signaling, and genotoxicity, respectively (13–15). These differences should be considered when evaluating how distinct pathogens engage host innate immune regulation during tumor development.

### 2.2 PAMP-driven activation of innate immune inflammatory pathways

A critical initiating event in pathogen-induced tumor-associated inflammation is the recognition of PAMPs by the host innate immune system. Gastrointestinal epithelial cells, dendritic cells, macrophages, and other myeloid cells express a wide range of PRRs that sense bacterial, viral, and other pathogen-derived molecular components, thereby initiating local inflammatory responses (54, 55).

Bacterial LPS, lipoproteins, flagellin, and CpG DNA are recognized by TLR4, TLR2, TLR5, and TLR9, respectively (18, 56). Intracellular bacterial components trigger NOD-like receptors (NLRs) and the NLRP3 inflammasome (57), whereas viral RNA and DNA are detected via the RIG-I/MDA5 and cGAS-STING pathways, respectively (58). These signals converge on NF-κB, MAPK, and IRF transcriptional programs, driving the expression of inflammatory mediators such as IL-1β, IL-6, TNF, CXCL8, CCL2, and type I IFNs. This cascade amplifies local inflammation and remodels the tissue microenvironment (59).

During acute infection, these inflammatory responses promote pathogen clearance, limit tissue damage, and support subsequent repair. However, when pathogens persistently colonize or chronically stimulate the mucosa, inflammation can shift from a transient protective response to chronic pathological inflammation. Persistent activation of the NF-κB and STAT3 signaling drives aberrant epithelial proliferation, enhances anti-apoptotic signaling, and promotes angiogenesis, stromal remodeling, and an immunosuppressive microenvironment (30, 60). Meanwhile, reactive oxygen species (ROS) and reactive nitrogen species (RNS) generated during inflammation further exacerbate DNA damage, mutagenesis, and genomic instability, thereby increasing the risk of tumorigenesis (61, 62).

Importantly, pathogens do not merely passively trigger inflammatory pathways; they also actively shape the nature and direction of the inflammatory response. For example, *H. pylori* reprograms gastric epithelial signaling via multiple virulence factors (63, 64); *F. nucleatum* promotes the accumulation of immunosuppressive myeloid cells while weakening antitumor immunity (15, 65, 66); and EBV enables tumor immune escape through modulation of antiviral immunity, antigen presentation, and immune checkpoint pathways (67). The resulting chronic inflammatory state keeps pro-proliferative, reparative, and pro-angiogenic signals active, while weakening effective pathogen clearance and immune surveillance, thereby creating favorable conditions for gastrointestinal tumor initiation and progression.

### 2.3 Imbalanced antimicrobial responses and the transition from chronic inflammation to a tumor-promoting state

Tumor-associated inflammation has traditionally been viewed as a process in which “inflammation promotes cancer.” However, increasing evidence indicates that tumor outcome is determined not simply by the intensity of inflammation, but by the dynamic imbalance among distinct phases of the inflammatory response.

A complete antimicrobial response typically consists of four sequential stages: pathogen recognition, inflammatory amplification, pathogen clearance, and tissue repair (25). Under physiological conditions, inflammatory signals rapidly recruit neutrophils, macrophages, natural killer cells, and effector T cells, which cooperate to eliminate pathogens. Subsequently, pro-resolving mediators, inflammatory negative-feedback mechanisms, and tissue repair programs act to terminate inflammation and restore local tissue homeostasis (68, 69). In gastrointestinal tumor-associated infection, however, pathogens or pathobionts often occupy specialized ecological niches, such as the mucus layer, gastric glands, intestinal crypts, biofilms, and hypoxic tumor microenvironments. These niches allow pathogens to evade complete eradication and maintain persistent inflammatory stimulation (70, 71).

Persistent pathogen-derived stimulation sustains the inflammatory response in a chronic, low-grade state. On the one hand, the IL-6/STAT3, TNF/NF-κB, and IL-17 signaling continuously promote epithelial proliferation, tissue repair, and microenvironmental remodeling. On the other hand, pathogens and the tumor microenvironment induce the accumulation of immunosuppressive cells, upregulate immune checkpoint molecules, and activate inflammatory negative-regulatory mechanisms, thereby impairing effective pathogen clearance and antitumor immunity (25, 26). This chronic inflammatory state is therefore not merely an intensification of inflammation, but rather a pathological immune condition marked by persistent inflammatory activity and insufficient clearance capacity.

**Figure 1:**
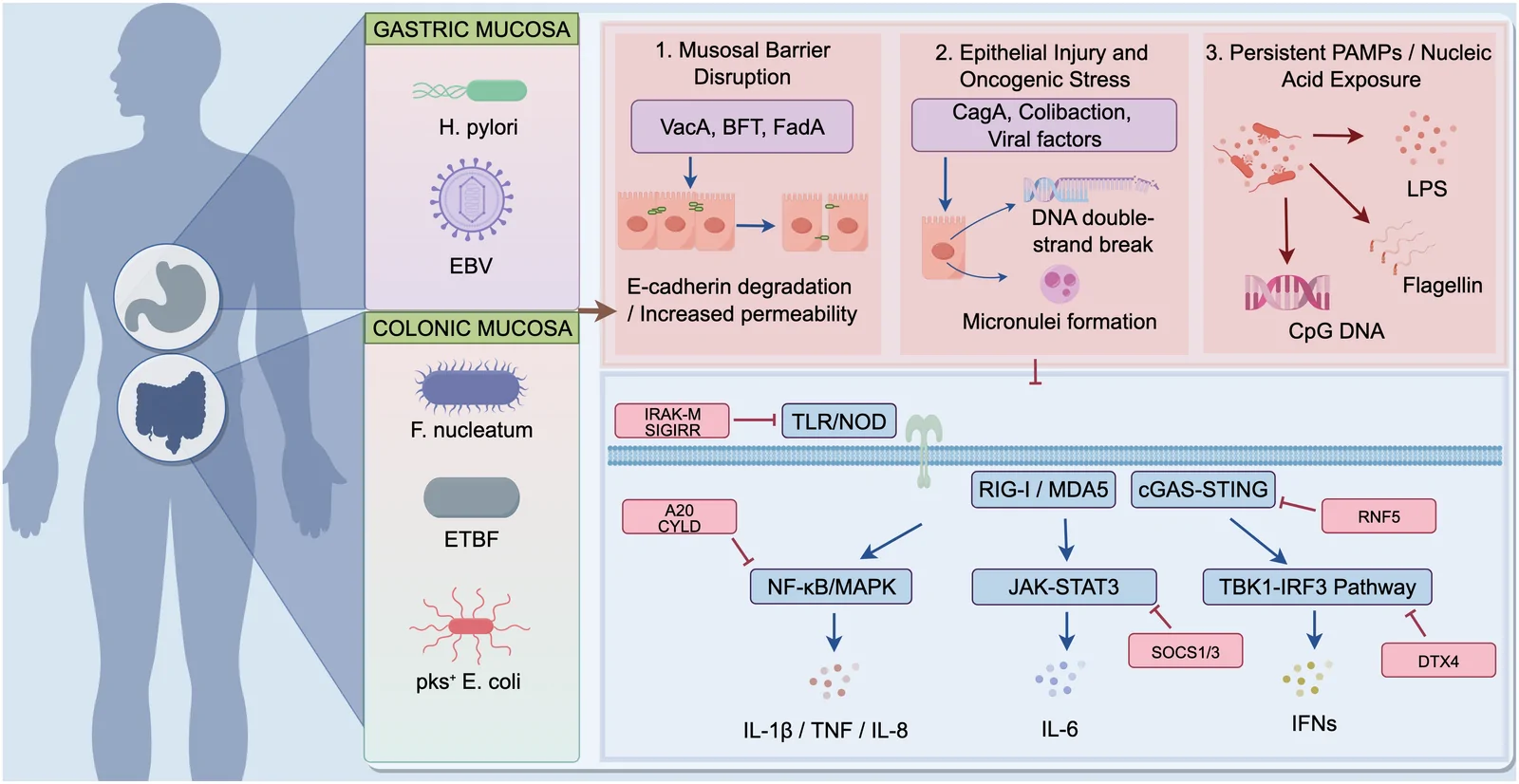
Persistent gastrointestinal pathogen colonization promotes mucosal injury and sustained innate immune signaling under the control of host negative regulatory networks. In the gastric mucosa, *H. pylori* and EBV persistently colonize the gastric epithelial niches, whereas *F. nucleatum*, ETBF, and pks-positive *E. coli* may become enriched in the colonic mucosa or tumor-associated microbial environment. Pathogen-derived virulence factors drive mucosal barrier disruption, epithelial injury, and oncogenic stress via E-cadherin disruption, increased permeability, DNA damage, and micronucleus formation. Persistent exposure to PAMPs and nucleic acids, including LPS, flagellin, CpG DNA, and viral nucleic acids, activates TLR/NOD, RIG-I/MDA5, and cGAS-STING signaling, resulting in the activation of NF-κB/MAPK, JAK-STAT3, and TBK1-IRF3 and the production of inflammatory cytokines and type I IFNs. These pathways are simultaneously restrained by host negative regulators, including IRAK-M/SIGIRR at TLR-associated signaling, A20/CYLD at NF-κB/MAPK signaling, SOCS1/3 at JAK-STAT3 signaling, RNF5 at cGAS-STING signaling, and DTX4 at the TBK1-IRF3 axis. The balance between persistent pathogen-derived stimulation and host negative regulation results in sustained yet incompletely resolved mucosal inflammation. Image created by figdraw.com.

Meanwhile, interactions among pathogens, host genetic background, mucosal barrier function, and the local metabolic environment collectively determine the magnitude, duration, and tissue consequences of post-infectious inflammation. Genetic variants involving TLR, NOD, STAT3, DNA repair, and barrier-related pathways can alter pathogen sensing, inflammatory signaling, and repair efficiency, contributing to individual susceptibility to chronic gastrointestinal inflammation and tumorigenesis (72–75). Similarly, microbiota-derived metabolites (bile acids, short-chain fatty acids, and tryptophan metabolites) influence epithelial barrier function, immune cell differentiation, and tissue homeostasis via FXR/TGR5, GPR41/GPR43, histone deacetylase inhibition, and AhR-dependent pathways (76–78). Over time, chronic inflammation not only increases DNA damage and mutational burden, but also imposes continuous immune selection pressure. This favors the clonal expansion of mutant cells that carry survival or proliferative advantages, ultimately driving tumor development (79) (**Figure 1**).

Taken together, pathogen-associated inflammation is not simply a process of inflammatory intensification. Rather, it is a dynamic evolutionary process shaped by persistent pathogen-derived stimulation, host immune responses, tissue repair programs, and the local microenvironment. In essence, protective inflammation fails to resolve and restore homeostasis. Instead, it persists and gradually evolves into chronic pathological inflammation that promotes tumor initiation, immune escape, and clonal selection.

## 3 Pathogen immune evasion and host innate immune negative regulation in gastrointestinal tumors

The innate immune system senses gastrointestinal pathogens via PRRs, while host negative-feedback mechanisms restrain excessive inflammation and maintain mucosal homeostasis (80). Here, “innate immune negative regulatory networks” refers to host-derived inhibitors of PRR- and IFN-associated signaling, including A20, CYLD, SOCS proteins, IRAK-M, SIGIRR, NLRX1, RNF5, TRIM29, USP21, and DTX4 (80–91). This host-derived negative regulation is distinct from pathogen-mediated immune evasion and downstream adaptive immune suppression. Although these mechanisms are distinct from pathogen-mediated immune evasion and downstream checkpoint suppression, they can converge to sustain pathogen persistence, impair antigen presentation, and weaken immune clearance. Their effects are also stage- and cell-type-dependent and should not be viewed as uniformly tumor-promoting.

The strength of pathogen-specific evidence varies among individual regulators. Direct evidence supports *H. pylori*-induced A20, SOCS3, and IRAK-M expression and *F. nucleatum* Fap2–TIGIT-mediated immune suppression (92–95). By contrast, RNF5, NLRX1, and TRIM29 are discussed mainly as established regulators of PRR or IFN signaling, because their direct roles in pathogen-associated gastrointestinal tumors require further validation (96–98).

### 3.1 Pathogen evasion of immune recognition in persistent colonization

The long-term persistence of gastrointestinal pathogens depends primarily on their ability to evade host innate immune recognition. Under physiological conditions, recognition of PAMPs by PRRs triggers rapid inflammatory responses and translates pathogen-derived signals into clearance programs. However, many gastrointestinal pathogens can reduce PAMP immunogenicity, modify PAMP structures, form biofilms, enhance adhesion, or interfere with nucleic acid sensing pathways. These strategies place the host in a state of insufficient or incomplete sensing. In this setting, pathogen-derived stimulation persists, but the resulting immune response remains inadequate for clearance, thereby facilitating low-grade inflammation, barrier injury, and aberrant tissue repair.

*H. pylori* provides a representative example of this process. Its LPS exhibits low immunogenicity, and structural modifications of lipid A can attenuate TLR4-mediated inflammatory recognition (99, 100). Its flagellin also shows weak TLR5-stimulatory or immune-evasive properties, making it difficult for the host to mount an effective clearance response via classical TLR4/TLR5 pathways (101). Consequently, *H. pylori* infection frequently does not manifest as a short-lived, intense acute inflammatory response. Instead, it more commonly generates low-grade, persistent, and incompletely resolved gastric mucosal inflammation. Over time, this state of “insufficient recognition despite persistent stimulation” creates a chronic inflammatory foundation for gastric mucosal atrophy, intestinal metaplasia, dysplasia, and gastric cancer development (102, 103).

Colorectal cancer-associated bacteria can also promote persistent colonization by altering host sensing thresholds and mucosal localization. *F. nucleatum* relies on adhesins such as FadA to enhance binding to intestinal epithelial and tumor cells, thereby facilitating its enrichment and persistence within tumor tissues (104, 105). ETBF secretes BFT, which disrupts E-cadherin-mediated epithelial barrier integrity and exposes the mucosal microenvironment to sustained bacterial components and inflammatory stimuli. BFT also activates β-catenin, NF-κB, STAT3, and Th17/IL-17-related pathways, driving persistent mucosal inflammation (106–109). pks-positive *E. coli*, particularly under barrier disruption and dysbiosis, can continuously expose the mucosa to PAMPs (e.g., LPS, flagellin, and CpG DNA), thereby activating TLR4, TLR5, TLR9, and MyD88-NF-κB/MAPK signaling (110–112). Its genotoxin colibactin induces DNA double-strand breaks, micronucleus formation, and chromosomal instability (13, 113–115). These DNA damage events may serve as potential danger signals for DNA-sensing pathways (e.g., cGAS-STING), although the causal links among persistent pathogen colonization, STING activation, and tumor promotion remain to be further validated.

DNA and RNA sensing systems are also critical components of antimicrobial immune surveillance, in addition to TLR-mediated sensing. The cGAS-STING and RIG-I/MDA5-MAVS pathways detect pathogen-derived nucleic acids, mitochondrial nucleic acids, as well as danger signals released during DNA damage. These pathways induce type I IFNs, CXCL9, CXCL10, and IL-12, which in turn promote dendritic cell maturation, antigen cross-presentation, and clearance mediated by NK cells and CD8^+^ T cells (115–118). However, nucleic acid sensing pathways are themselves subject to multilayered negative regulation. Some regulators, including RNF5, TRIM29, USP21, and NLRC3, attenuate cGAS-STING signaling by affecting STING stability, STING-TBK1 complex formation, or IRF3 activation; by contrast, DTX4 suppresses type I IFN production via promoting TBK1 ubiquitination and degradation (91, 96, 119, 120). A20, RNF125, LGP2, and NLRX1 restrict RIG-I/MDA5-MAVS signalosome formation or promote degradation of key adaptor proteins, thereby suppressing the output of RNA-sensing pathways (97, 121–125). In the setting of chronic pathogen stimulation and the tumor microenvironment, danger signals may persist, yet their conversion into type I IFN responses and cytotoxic immune priming may be attenuated by negative regulatory networks.

EBV-positive gastric cancer further illustrates the tension between nucleic acid sensing and immune escape. Latent EBV infection continuously supplies viral nucleic acids, viral non-coding RNAs, and IFN-related stimuli, driving prominent immune cell infiltration within tumor tissues (126, 127). However, viral miRNAs, latency-associated genes, PD-L1 upregulation, and host negative regulatory network remodeling may collectively impair antigen presentation, IFN signaling, and cytotoxic immune function. This can generate a state characterized by marked immune infiltration coupled with insufficient effective clearance (52, 128). Thus, pathogen evasion of immune recognition should not be equated with complete avoidance of inflammation. Rather, they may maintain inflammatory recognition at a level insufficient for clearance but sufficient to drive chronic tissue injury and tumor-promoting signals (**Table 1**). Accordingly, pathogen immune evasion acts as an upstream driver that sustains persistent stimulation, whereas host negative regulatory pathways control how efficiently these signals are converted into IFN production, antigen presentation, and cytotoxic immune priming.

**Table 1:** Negative regulators of innate immunity in pathogen-associated gastrointestinal tumors and their immunological consequences.

Negative regulator	Main targeted pathway	Immunological consequence in gastrointestinal tumors	References
A20/TNFAIP3	TLR-NF-κB; RIG-I/MAVS	Limits NF-κB and antiviral signaling, reduces type I IFN production and antigen presentation, and may promote pathogen persistence and immune tolerance.	(86, 123, 124)
CYLD	TLR/TNF-NF-κB; MAPK	Restrains ubiquitin-dependent inflammatory signaling. Its sustained activity may dampen pathogen clearance, whereas loss of CYLD may enhance tumor-promoting inflammation, indicating context-dependent effects.	(82, 87, 88)
SOCS1/SOCS3	JAK-STAT; TLR-cytokine crosstalk	Suppresses cytokine and IFN signaling, limits macrophage and dendritic cell activation, and favors an immunosuppressive microenvironment.	(83, 129, 130)
IRAK-M	TLR-MyD88-IRAK	Induces a hyporesponsive myeloid state, reduces PAMP-induced macrophage and dendritic cell activation, and contributes to ineffective pathogen clearance.	(84, 132)
SIGIRR	TLR/IL-1R	Attenuates TLR/IL-1R-driven inflammatory amplification. While maintaining barrier homeostasis, persistent upregulation may weaken pathogen sensing and immune surveillance.	(85, 89)
NLRX1	RIG-I/MDA5-MAVS; NF-κB	Dampens antiviral and inflammatory signaling, reducing type I IFN responses and cytotoxic immune priming in chronic pathogen- or virus-associated settings.	(90, 125, 133, 134)
RNF5	cGAS–STING	Promotes STING degradation or limits STING stability, thereby reducing DNA damage- or pathogen DNA-induced type I IFN responses and dendritic cell/T-cell activation.	(96)

### 3.2 Pathogen-mediated suppression of innate immune effector cells and clearance failure

After long-term colonization of the mucosal niche, pathogens can further impair the clearance capacity of innate immune effector cells. Macrophages, neutrophils, natural killer (NK) cells, and dendritic cells normally coordinate through pathogen phagocytosis, microbial killing, antigen presentation, inflammatory amplification, and cytotoxic activity. However, under chronic pathogen stimulation and within the tumor microenvironment, these cells frequently undergo functional reprogramming. Their phagocytic and antimicrobial capacities decline, pro-inflammatory and immunosuppressive phenotypes coexist, cytotoxic responses become insufficient, and antigen presentation efficiency diminishes (135). Consequently, persistent inflammatory signals fail to translate into effective pathogen clearance or antitumor immunity, and the local microenvironment progressively shifts toward immune tolerance and tumor immune escape.

Macrophages serve as central regulators linking pathogen clearance, inflammatory control, and tissue repair. During acute infection, macrophages restrict pathogen expansion through phagocytosis, lysosomal killing, ROS/RNS production, and the release of inflammatory cytokines (136, 137). During chronic *H. pylori* infection, CagA, VacA, and other secreted factors can reprogram signaling in gastric epithelial cells, macrophages, and dendritic cells. These events induce negative regulatory molecules (e.g., SOCS3 and IRAK-M), thereby limiting IL-12 production, antigen presentation, and T-cell activation (83, 86, 94, 132, 138–140). As inflammation persists, macrophages gradually transition from a pathogen-clearing phenotype toward a tumor-associated macrophage (TAM) or an M2-like phenotype. These cells produce immunosuppressive molecules (e.g., IL-10, TGF-β, ARG1, and PGE2), while also promoting tissue repair, angiogenesis, and tumor cell survival (141–143). Thus, macrophage remodeling not only impairs pathogen clearance but also facilitates the conversion of chronic inflammation into a repair-oriented response that supports tumor progression.

Neutrophils also contribute to the persistence of chronic gastrointestinal inflammation. Under physiological conditions, neutrophils eliminate pathogens via phagocytosis, degranulation, ROS production, and neutrophil extracellular trap (NET) formation (144, 145). However, in the presence of persistent PAMP stimulation, activation of the STAT3/IL-17 axis, and tumor-associated chemokines, neutrophil function can become dysregulated. On the one hand, sustained neutrophil recruitment drives tissue injury, oxidative stress, and extracellular matrix remodeling. On the other hand, impaired antimicrobial activity and defective inflammatory resolution prevent effective clearance (146, 147). The BFT-STAT3/IL-17 inflammatory axis induced by ETBF can sustain myeloid-cell and neutrophil-associated inflammatory responses, shifting mucosal inflammation from a protective antimicrobial response toward tumor-promoting inflammation (148). DNA damage induced by pks-positive *E. coli* can trigger cellular senescence and a senescence-associated secretory phenotype, including increased IL-6 and MMP-3 production (149). In this context, neutrophil-associated inflammation no longer functions primarily in pathogen elimination, but rather contributes to tissue damage, inflammatory persistence, and the establishment of a tumor-promoting microenvironment.

NK cells and T cells are key effector populations in antiviral and antitumor immunity. Their production of IFN-γ and cytotoxic killing activity restrict the expansion of infected and transformed cells (150, 151). Pathogens and the tumor microenvironment can weaken these effector functions by promoting inhibitory receptor signaling, immune checkpoint pathways, and immunosuppressive mediators. The Fap2 protein of *F. nucleatum* can bind the inhibitory receptor TIGIT on T cells and NK cells, thereby dampening cytotoxic immune responses (95). In chronic gastrointestinal inflammation and the tumor microenvironment, IL-10, TGF-β, PGE2, and PD-1/PD-L1 signaling further inhibit IFN-γ production and cytotoxic activity in NK cells, enabling pathogen-infected cells and transformed cells to evade immune clearance more effectively (152).

Suppression of dendritic cell function represents a critical step linking innate immune defects to adaptive immune failure. TLR, STING, and RIG-I/MDA5 pathways normally promote dendritic cell maturation, costimulatory molecule expression, and IL-12 production, which in turn supports CD8^+^ T cell-mediated antitumor immunity. SOCS, IRAK-M, and A20 can inhibit TLR-induced IL-12 production and antigen presentation (83, 94, 123, 129–131, 139, 140). In parallel, RNF5, TRIM29, USP21, DTX4, RNF125, and NLRX1 can limit STING, TLRs or RIG-I/MDA5 pathway output, leading to insufficient type I IFN, CXCL9, and CXCL10 production (96, 97, 120, 133, 153–155). *H. pylori* can also suppress dendritic cell maturation, promote Treg skewing, and enhance IL-10/TGF-β-mediated immunosuppressive signaling, thereby fostering long-term immune tolerance and compromised antigen clearance in the gastric mucosa (156, 157). Therefore, even in the presence of persistent pathogen stimulation, DNA damage, and cell death signals within gastrointestinal tumor tissues, these danger signals may not be efficiently converted into dendritic cell-mediated antigen cross-presentation and cytotoxic T cell priming.

**Figure 2:**
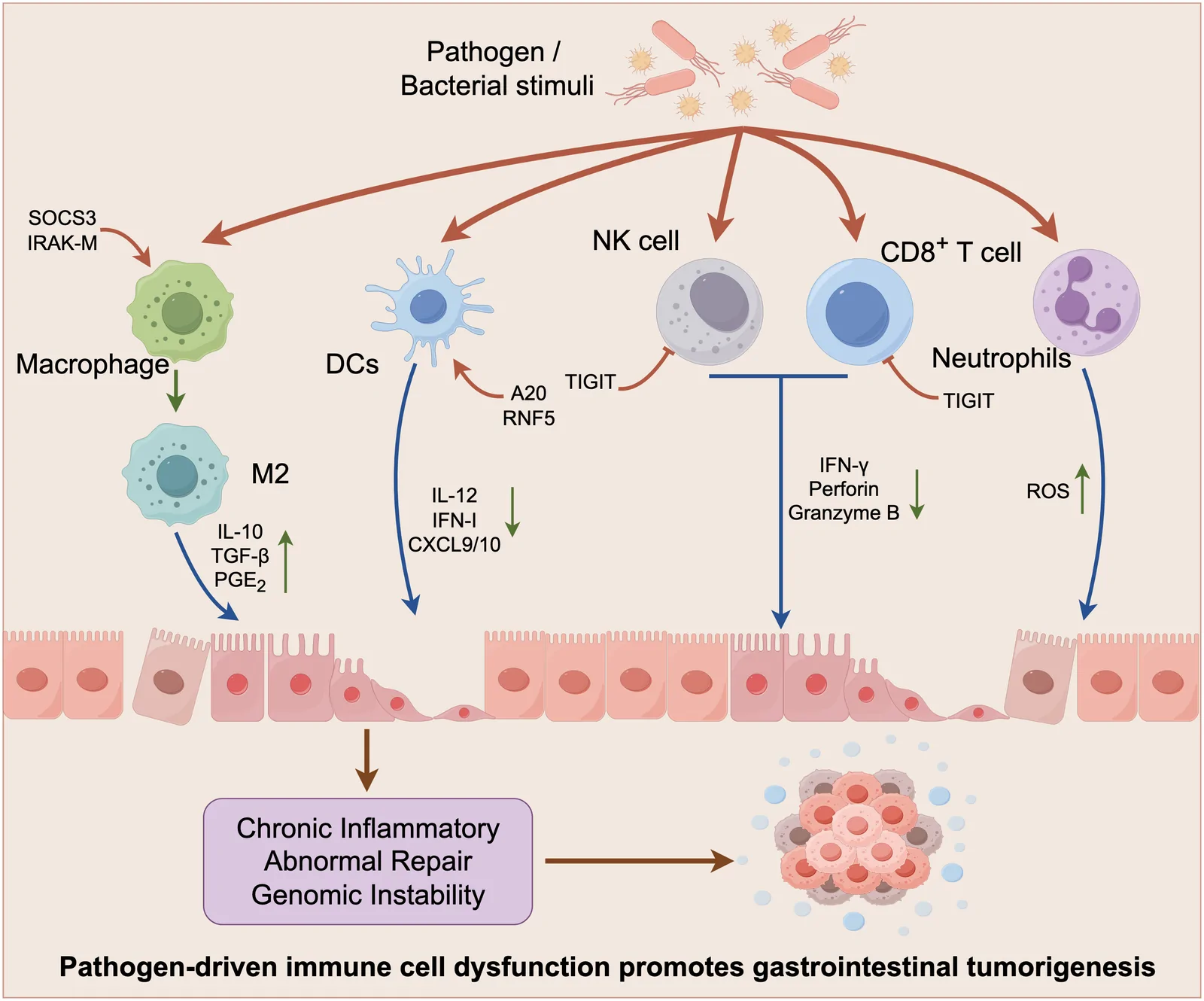
Pathogen-driven immune cell function remodeling contributes to impaired immune clearance and gastrointestinal tumorigenesis. Under chronic stimulation by gastrointestinal pathogens or pathobionts, local immune cells undergo functional reprogramming and gradually shift from effective antimicrobial responses toward persistent inflammation and immune suppression. SOCS3- and IRAK-M-associated negative regulation in macrophages is accompanied by the development of M2-like or tumor-associated phenotypes and increased production of IL-10, TGF-β, and PGE2. In dendritic cells, negative regulatory mechanisms involving A20 and RNF5 are associated with impaired activation and reduced IL-12, type I IFN, and CXCL9/10 production, thereby weakening antigen presentation and downstream cytotoxic immune priming. In parallel, inhibitory receptor signaling (such as TIGIT) suppresses NK- and CD8+ T-cell effector functions, resulting in reduced expression of IFN-γ, perforin, and granzyme B Persistent neutrophil activation enhances ROS production and further exacerbates epithelial injury and tissue remodeling. Together, these alterations promote chronic inflammation, abnormal tissue repair, genomic instability, and ultimately gastrointestinal tumorigenesis. Green upward arrows indicate increased molecules or functions, green downward arrows indicate decreased molecules or functions, blue arrows indicate immune-cell effects on the epithelial barrier, and brown arrows indicate pathogen-derived stimulation and downstream tumor-promoting processes. Image created by figdraw.com.

Overall, pathogen-associated immune dysfunction in gastrointestinal tumors reflects the functional convergence of pathogen immune evasion, host innate immune negative-feedback regulation, and downstream effector immune suppression, reorienting the inflammatory response. By evading immune recognition, pathogens establish persistent colonization and further impair macrophage-mediated microbial killing, dendritic cell antigen presentation, NK/T-cell cytotoxicity, and neutrophil-mediated clearance. Consequently, the local immune response in the gastrointestinal tract shifts from a protective “recognition-clearance-repair” program to a chronic state of persistent stimulation, impaired effector clearance, and immune tolerance. The coexistence of sustained inflammation and defective clearance provides a critical foundation for pathogen persistence, aberrant tissue repair, immune escape, and gastrointestinal tumor progression (**Figure 2**).

### 3.3 Bidirectional roles of innate immune negative regulators in gastrointestinal tumorigenesis

Importantly, innate immune negative regulators are not simply tumor-promoting factors. Their functions depend heavily on disease stage, cellular context, and the inflammatory environment. During infection or chronic mucosal inflammation, negative-feedback regulators can restrain excessive PRR, NF-κB, JAK–STAT, and IFN signaling, thereby limiting tissue-damaging inflammation. For example, *H. pylori* infection induces A20 in gastric epithelial cells and SOCS3 in dendritic cells, whereas IRAK-M and SIGIRR constrain TLR-associated inflammatory responses in both gastric and intestinal tissues (92–94). However, these regulatory mechanisms may simultaneously attenuate antimicrobial or antigen-presenting functions under persistent stimulation, ultimately impairing pathogen clearance.

These infection-associated effects should be distinguished from the roles of the same regulatory networks in later stages of carcinogenesis. Genetic, epigenetic, and post-transcriptional alterations can reduce or functionally disrupt negative regulators that normally constrain oncogenic inflammatory and survival pathways. In gastric cancer, SOCS1 promoter hypermethylation and reduced SOCS1/3 expression have been reported (158, 159), while CYLD expression is reduced in human colon carcinomas (160). In colorectal cancer, SIGIRR function can also be disrupted by a dominant-negative isoform with defective membrane localization (85, 161). Conversely, not all negative regulators are lost during tumor progression; in colorectal cancer, tumor-cell IRAK-M is upregulated and promotes tumor growth by weakening antimicrobial defense and stabilizing STAT3 (84). Thus, innate immune negative regulators do not follow a single trajectory from infection to malignancy. Their contribution should instead be interpreted according to disease stage, cellular localization, molecular integrity, and pathway context, rather than being viewed as uniformly “co-opted” by pathogens or uniformly tumor suppressive.

## 4 Stratified intervention strategies from pathogen control to innate immune reprogramming

Pathogen-associated inflammation in gastrointestinal tumors is not simply a process of immune activation, but rather the result of persistent pathogen stimulation, innate immune negative regulation, and tumor ecosystem remodeling. Its defining feature is the coexistence of sustained inflammation and insufficient pathogen clearance, antigen presentation, and cytotoxic immunity—a combination that may manifest as chronic non-resolving inflammation, myeloid suppression, and tumor immune escape (26, 27, 135). Therefore, future therapeutic strategies should not merely amplify inflammatory responses. Instead, they should aim to restore the complete “recognition-presentation-killing-clearance” axis while curbing pathogen-derived stimulation and limiting pathological inflammation (162, 163). To achieve this goal, pathogen control, barrier repair, innate immune reactivation, myeloid inflammation remodeling, and immune checkpoint-based combination therapy should be integrated into a unified, stratified intervention framework.

### 4.1 Shifting therapeutic goals from enhancing inflammation to restoring the clearance phase

Current intervention strategies for pathogen-associated gastrointestinal tumors can be broadly grouped into two categories. The first category aims to reduce persistent pathogen-derived stimulation and reshape the microbial ecosystem. Among these approaches, *H. pylori* eradication is already an established clinical strategy for gastric cancer prevention, while direct targeting of *F. nucleatum*, ETBF, or pks-positive *E. coli*, along with microbiota modulation, fecal microbiota transplantation, phage therapy, and engineered bacteria, remains investigational or largely preclinical in this setting (164–167). The second category seeks to reactivate suppressed innate immune clearance functions, through approaches such as STING agonists, TLR agonists, RLR pathway agonists, and strategies targeting negative regulatory nodes of innate immunity. Most of these approaches remain at preclinical or early clinical stages, rather than being established treatments for pathogen-associated gastrointestinal cancers (168–170).

The shared objective of these strategies is not to intensify inflammation in the tumor microenvironment, but to shift it from a state of “inflammation without effective clearance” into an immune state that is recognizable, presentable, and cytotoxic. STING agonists may enhance type I IFN production, promote dendritic cell maturation, and facilitate CD8^+^ T cell priming (171–173). TLR3, TLR7/8, and TLR9 agonists can function as local immune adjuvants, enhancing antigen presentation, dendritic cell activation, and T cell immune priming (174–176). Targeted release of selected negative regulatory nodes may restore TLR, STING, or RIG-I/MDA5 pathway output, enabling pathogen-derived nucleic acids, DNA damage, and therapy-induced danger signals to be converted into effective immune responses (96, 98, 125).

However, innate immune reprogramming faces a clear therapeutic window challenge. Excessive activation of STING or TLR signaling may cause cytokine toxicity, intestinal inflammation, or barrier injury, whereas insufficient activation may merely add to the inflammatory background without truly restoring pathogen clearance or antitumor immunity. Therefore, the development of innate immune agonists should carefully consider dose, delivery route, local activity, and indication-based patient selection (177–179). Similarly, negative regulatory molecules are broadly involved in barrier homeostasis, antimicrobial immunity, and tissue repair, and are not tumor cell-specific. Future strategies should therefore avoid long-term systemic release of immune “brakes.” Instead, they should emphasize short-term, local, and cell-type-directed immune reprogramming, with a focus on restoring the clearance axis involving dendritic cells, macrophages, NK cells, and CD8^+^ T cells.

Safety is a major consideration when targeting host negative regulatory nodes, given their essential roles in intestinal homeostasis. Systemic A20 deficiency leads to severe inflammatory disease, cachexia, and premature death, while combined loss of A20 in intestinal epithelial and myeloid cells drives ileitis, severe colitis, epithelial injury, dysbiosis, and colorectal tumorigenesis in aged mice (180, 181). SOCS1 also plays an important role in limiting intestinal inflammation, with its deficiency exacerbating experimental colitis and promoting spontaneous colorectal carcinogenesis in mice via excessive IFN-γ/STAT1 signaling (129, 182). Accordingly, therapeutic strategies targeting these pathways must carefully balance the benefits of enhanced antigen presentation and antimicrobial or antitumor effector activity against the risk of excessive mucosal inflammation, epithelial injury, barrier disruption, and IBD-like pathology.

### 4.2 Patient stratification based on pathogen burden, pathway status, and inflammatory type

Pathogen-driven negative regulation of innate immunity is highly heterogeneous. Patients may vary substantially in pathogen burden, PRR pathway integrity, myeloid-driven inflammatory intensity, negative regulatory network activity, and antigen presentation capacity. Mechanism-based stratification is therefore required before treatment, rather than simply deciding whether immunity should be “enhanced” (27, 183).

The first group includes patients with high pathogen burden and preserved PRR pathway responsiveness, but insufficient clearance. Examples include *H. pylori*-positive gastric precancerous lesions and colorectal cancers enriched with *F. nucleatum*. For these patients, pathogen control and barrier repair should be prioritized to reduce persistent sources of stimulation, with innate immune agonists or checkpoint therapy considered based on the local immune state (164–166). If pathogen-derived stimulation is not controlled, simply enhancing inflammation may further exacerbate tissue injury and tumor-promoting inflammation.

The second group includes patients with structurally intact STING, TLR, or RIG-I/MDA5 axes that are suppressed by myeloid inhibition or negative regulatory networks. These tumors may respond to local STING or TLR agonists combined with PD-1/PD-L1 blockade, radiotherapy, or DNA damage-inducing therapy (175, 184). The rationale is to boost tumor antigen release, enhance dendritic cell cross-presentation, and promote T cell infiltration, thereby converting therapy-induced danger signals into sustained antitumor immunity.

The third group consists of tumors driven by pathological inflammatory axes such as IL-6/STAT3, IL-1β, CXCL-CXCR2, or CSF1R-mediated myeloid inflammation. Although these tumors may appear immune-infiltrated, their inflammation is largely sustained by tumor-promoting myeloid cells, TAMs, MDSCs, and inflammatory fibroblasts (185–187). In these patients, simply activating the TLR or STING signaling may further amplify pathological inflammation. A more rational approach may be to first block tumor-promoting inflammatory axes (e.g., IL-1β, IL-6, CSF1R, or CXCR2) to reduce myeloid suppression, and then consider immune activation (188, 189).

The fourth group includes patients with genetic or epigenetic defects in key immune pathways. For example, when STING, antigen presentation pathways, JAK/STAT signaling, or IFN response modules are irreversibly impaired, activating PRRs alone may be ineffective (190–192). In such settings, bypass strategies such as oncolytic viruses, cellular therapy, bispecific antibodies, or microbiome-assisted approaches may be considered to improve checkpoint therapy responses (193, 194). Thus, future therapeutic decisions should not simply ask whether immunity should be enhanced. Instead, they should define which factor—the pathogen-derived stimulus, the negative regulatory node, the cell type, or the disease stage—prevents entry into an effective clearance phase.

### 4.3 Establishing companion diagnostics and combination treatment systems

Future preclinical and clinical studies should not rely solely on tumor volume or survival as endpoints. Pathogen status, innate immune function, and tissue ecology should be integrated into a unified evaluation system. For therapies targeting STING, TLR, or RIG-I-like pathways, it will be necessary to evaluate pathogen burden and spatial localization, the balance between type I IFN and NF-κB output, dendritic cell cross-presentation capacity, NK cell cytotoxicity, the degree of myeloid suppression, T cell infiltration status, barrier damage, and systemic toxicity (195–197). The translational value of a treatment can only be assessed by determining whether it truly restores the “recognition-presentation-killing-clearance” axis (163, 198).

A companion diagnostic system may be established at four levels. Pathogen-level assessment should cover *H. pylori* infection and virulence status, *F. nucleatum* burden, the pks island, the BFT gene, and EBV status (45, 164, 165, 167, 199). At the host pathway level, the expression and functional status of negative regulatory factors and the RIG-I/MDA5 axis should be evaluated (172, 183). Functional-level evaluation should focus on type I IFN, CXCL9/10, IL-6/STAT3, CXCL1/2/8-CXCR2, antigen presentation genes, and myeloid suppression scores (188, 189, 198). Spatial-level analyses should assess whether pathogen-enriched regions colocalize with DNA damage, TAM/MDSC accumulation, T cell exclusion, barrier disruption, and aberrant repair (45, 195, 196). Integrating these parameters may help stratify patients into different therapeutic paths, including pathogen control, innate immune reactivation, myeloid remodeling, or checkpoint-based combination therapy.

Microbiome-based therapy should also move from simply “altering microbial abundance” toward restoring immune function. Antibiotics can reduce specific pathogen burdens, yet they may also disrupt beneficial commensals, increase antimicrobial resistance, and influence responses to immunotherapy (167). Probiotics, fecal microbiota transplantation, phage therapy, and engineered bacteria hold therapeutic potential, though their target species, colonization capacity, safety, and ecological consequences remain to be clearly defined (194, 200–202). For pathogen-driven negative regulation, the evaluation criteria for microbiome therapy should not be limited to the reduction of a given bacterium. Instead, the criteria should include restoration of TLR/STING/RIG-I pathway output, reduction of myeloid suppression, improvement of barrier function, and enhancement of T cell-mediated clearance (9, 163).

Overall, the interplay between pathogen-mediated immune evasion and host innate immune negative regulation provides a framework that integrates infectious disease, mucosal immunology, and tumor immunology in gastrointestinal cancer research. This framework explains why pathogens can induce inflammation yet escape clearance, and why immune cells may infiltrate tumors but fail to establish effective immune surveillance. It also explains why enhancing immunity does not necessarily restore clearance. A more promising therapeutic direction is to identify reversible states where inflammation persists without sufficient effector clearance. Therapeutic strategies should then restore effective immunity through stage-specific combinations of pathogen control, barrier repair, innate immune reactivation, myeloid remodeling, and checkpoint therapy.

## 5 Conclusions and perspectives

This review systematically summarizes the interplay among persistent gastrointestinal pathogen colonization, imbalanced innate immune recognition, remodeling of negative regulatory networks, and the formation of tumor-associated chronic inflammation. Although *H. pylori*, *F. nucleatum*, *ETBF*, pks-positive *E. coli*, and EBV differ in tissue distribution, virulence factors, and immune regulatory strategies, they are all capable of persisting within specific gastrointestinal mucosal niches. Through sustained PAMP stimulation, barrier injury, DNA damage, impaired antigen presentation, and suppression of effector cell function, these pathogens shift the local immune microenvironment away from protective antimicrobial immunity toward chronic inflammation and immune tolerance. The central concept of this review is that pathogen-associated inflammation in gastrointestinal tumors should not be viewed simply as “enhanced inflammation that promotes cancer”. Rather, its more critical pathological feature is the coexistence of persistent inflammation and insufficient immune clearance. In other words, pathogen-derived stimulation persists and inflammation is not fully resolved, yet these signals fail to translate into effective dendritic cell antigen presentation, NK/T cell-mediated killing, and pathogen clearance. This ultimately establishes a tumor-promoting cycle of “persistent stimulation-inefficient clearance-aberrant repair-immune escape”.

Based on this concept, pathogen-driven negative regulation of innate immunity offers an integrative framework for understanding how infection, chronic inflammation, and immune escape intersect in gastrointestinal tumors. Its conceptual significance lies in shifting the research focus from merely measuring elevated inflammatory mediators to determining whether inflammatory responses actually progress to an effective clearance phase. Future studies should further clarify the spatial localization of various pathogens within tumor tissues, the cell-type-specific functions of negative regulatory molecules, and the dual roles of the TLR, STING, and RIG-I/MDA5 pathways at different stages of tumor development. Therapeutically, strategies should not simply aim to enhance immunity or suppress inflammation. Instead, stratified interventions should be guided by pathogen burden, barrier status, innate immune pathway activity, myeloid suppression, and T cell functional state. Rational combinations of pathogen control, barrier repair, innate immune reactivation, myeloid inflammation remodeling, and immune checkpoint therapy could restore the “recognition-presentation-killing-clearance” axis in gastrointestinal tumors, offering new directions for the precision prevention and treatment of these cancers.
